# 

**Published:** 1911-04-01

**Authors:** 


### MEDICAL APPOINTMENTS, ETC.

The Election Committee has made the following appointments to the Honorary Staff of the North Staffordshire Infirmary: Mr. G. H. Sowry, M.D., B.S., L.R.C.P. Lond., F.R.C.S. Eng., and Mr. E. C. Myott, M.D., B.S., L.R.C.P. Lond., M.R.C.S. Eng., to be assistant physicians; Mr. H. Hartley, M.B., Ch.B. Vict., M.D., B.S. Lond., F.R.C.S. Eng., and Mr. E. E. Young, M.S., M.B. Lond, M.R.C.S. Eng., L.R.C.P. Lond., to be assistant surgeons; and Mr. A. H. John, M.B., B.S., L.R.C.P. Lond., M.R.C.S. Eng., to be additional medical officer for the Electrical Department.

Mrs. Gerald Peel, together with her daughter and sons, have promised £1,000 to endow a cot in the Manchester Children's Hospital, Pendlebury, to be named after the late Mr. Gerald Peel, a former vice-president and governor of the hospital for many years.

Mr. Henry Francis Brooks, of Meads, Eastbourne, lately in business as a timber merchant, has left the residue of his property, after certain bequests, to his wife for life, and subject thereto £250 each to the Victoria Cottage Hospital, Finchley, the Poplar Hospital for Accidents, the Children's Hospital, Great Ormond Street, W.C., and to the Princess Alice Memorial Hospital at Eastbourne.

The following have been appointed members of the Board of the Leicester Infirmary for the ensuing year: Mrs. R. F. Martin, Mr. L. F. Topham, Mr. George Green, and Mr. Albert Tyler, representing county subscribers; Mrs. Fielding Johnson, Mr. J. Collier, the Mayor of Leicester (Ald. W. W. Vincent), Mr. T. Fielding Johnson, jun., Mr. J. H. Corah, Mr. Alec Tyler, Mr. C. F. Oliver in place of his brother Mr. Geo. C. Oliver, the Deputy-Mayor (Councillor Chitham) *vice* Mr. Alfred Colson deceased, all representing the Borough of Leicester; the Rev. G. A. Tanner and Mrs. A. E. Codrington representing Rutlandshire. Mr. Francis Stene, J.P.D.L., has been re-elected treasurer.

### Gifts and Bequests.

Lord Sandhurst, treasurer of St. Bartholomew's Hospital, has received £1,000 from the Worshipful Company of Goldsmiths toward the reduction of the building debt.

Mr. Edward Critchell, of Lion House, Chichester, has left £50 to each of his executors and all other his property, subject to a life interest, to the Chichester Infirmary.

Mr. Edmund Grove, of Preston, Brighton, civil engineer, who left estate of the gross value of £56,993, has left £500 to the Brighton and Hove Hospital at Brighton.

Mr. Henry Barrell Bradley, of Folkestone, Kent, solicitor, has left £100 to the Victoria Hospital, Folkestone.

Mr. John Bonnett, M.A., of Cambridge, has left £200 to Addenbrooke's Hospital; and Mr. Eric Barff Charlesworth, of Lincoln's Inn and late of Newton Hall, Cambridge, has left £1,000 for the Children's Ward.

Lady Juliet Duff has received the following, among further contributions, for freeing Charing Cross Hospital from its mortgage debt: Mr. Joseph Shaw, K.C., £100; Mr. J. F. Mason, M.P., £25; Mr. W. J. Raphael, 15 guineas; the *Weekly Dispatch*, £10 4s.; Messrs. B. Burnet and Co. (Limited), £10; the London Library, five guineas; and £500 from the executors of Mlle. Louise Bellevue.

Mrs. Penelope Anne Britten, of Shermanbury Grange, Henfield, Sussex, who left estate of the gross value of £80,925, has bequeathed £500 to the Royal Sussex County Hospital.

Mr. H. O. Wills has given £5000 to the Bristol General Hospital.

Mr. Joseph Thomas, of Haverfordwest, has bequeathed £1000 to the Pembrokeshire and Haverfordwest Infirmary, to endow a "Joseph Thomas" bed.

The Committee for the Removal of King's College Hospital to South London has received £1,000 from the Goldsmiths' Company, this being their third donation to the fund.

The Goldsmiths' Company have sent a donation of £100 to the funds of the Royal Hospital for Incurables, Putney Heath, and have granted £250 to the Belgrave Hospital for Children.

Mr. Henry Veasey, F.R.C.S., of Aspley Guise, Beds, retired surgeon, who died on January 17, aged ninety-two, has left estate valued at £36,479 gross, with net personalty £32,865. He bequeathed £300 to the Royal Medical Benevolent Fund, and £200 to the Hunstanton Convalescent Home.

Sir Charles Rackham Gilman, of Stafford House, Norwich, has left £300 to the Norfolk and Norwich Hospital, £200 to the Jenny Lind Infirmary (Norwich), and £100 each to fifteen other Norfolk and Norwich charitable institutions.

An anonymous donation of £500 has been received by Mr. W. C. Bridgeman, M.P., on behalf of the Florence Nightingale Hospital.

Mr. James Archibald Duncan, ex-Liberal M.P. for Barrow-in-Furness 1890-2, has bequeathed £500 to the Perth New Infirmary.

Mr. Edward Filliter, of Hampstead, civil engineer, has left the residue of his estate upon trust as to one-fifth for his son for life, with remainder to his four daughters during their lives and the life of the survivor, and on the death of the survivor he left the one-fifth share as follows: One-third equally between the University College Hospital, London, and the Leeds General Infirmary.

### Jottings.

Princess Alexander of Teck will open in the summer a new ward containing fourteen beds at the Royal Sea Bathing Hospital, Margate.

A new clinic has been added to the Hospital of the Women's College, Pennsylvania, where women are undertaking a large proportion of surgical work.

Sir William P. Hartley has promised to build a new maternity hospital for Liverpool as soon as the endowment fund of £20,000 has reached two-thirds of that sum. The site will be on the Cambridge Street Almshouses.

The Hastings, St. Leonards, and East Sussex Hospital is to be removed to the corner of Bohemia Road and Cambridge Road. The site is being purchased by the local King Edward Memorial Fund, and has been long wished for.

The King has given permission for the two central ward blocks of the new King's College Hospital at Denmark Hill to be named the King Edward VII. block and the King George V. block.

Good progress is being made with the Sir William Cook memorial scheme at Birmingham. When the new home for consumptives at Romsley Hill is finished it will be handed over to the Birmingham Hospital Saturday Fund, to be managed and maintained by its committee.

Prince Alexander of Teck, who is chairman of the Appeal Committee of the Royal Waterloo Hospital for Children and Women, was patron of the two special performances of the Morality Play *Everyman*, which took place at Weybridge this week in aid of the hospital.

One of the schemes undertaken by Prince Alexander of Teck for the benefit of the Prince Francis of Teck Memorial Fund in aid of the Middlesex Hospital is an exhibition of the tiaras to be worn at the Coronation. Numerous promises to lend jewels have been received.

The latest scheme to help the Middlesex Hospital has been undertaken during this (all-British) shopping week, when £500 worth of Luce's eau de Cologne from Jersey was on sale at Harrod's. The saleswomen were popular actresses, and the sale was known as the Salon of Fragrance and Fair Women.

Among the old masters collected in the British Section of the Fine Arts at the International Exhibition at Rome is Gainsborough's great "John Eld, Esq.," the possession of which by the Staffordshire General Infirmary was made known in The Hospital of April 9, 1910 (p. 64). The institution has decided to part with the picture.

It was good to learn from the Lord Mayor of Newcastle, Sir W. H. Stephenson, that as a result of his motion in October last to set apart a room in the hospital for the religious services of the Church of England and of Nonconformity, the Newcastle Infirmary Chapel dispute is amicably lulled. Two solicitors are now conferring, and a joint report will be submitted to the High Court, which everyone hopes will lead to a settlement.

At Hinckley Cottage Hospital (Leicestershire) the admissions during the year numbered 150, and the out-patients 1,105 as against 704 in 1909. The receipts amounted to £777, of which the principal items were annual subscriptions £160 and workpeople's contributions £374. The expenditure was £736. The deficit at the beginning of the year of £157 has been reduced to £127. The Ladies' Committee reported that the nurse had attended 178 patients and made 4,190 visits.

### BUILDINGS CONTEMPLATED AND IN PROGRESS.

Arbroath.—Mr. Hugh Sairn, the architect, has reported on the proposed alterations at the Arbroath Infirmary, and has estimated the cost of them at £8,000. It will be remembered that Dr. Donald M'Intosh, M.V.O., of the Western Infirmary, Glasgow, prepared the sketch plans on which this estimate has been based. To build an entirely new infirmary on the present site, making use of the existing materials, it has been estimated would cost £14,000.

Brighton and Hove.—More than half of the £5,000 required for the King Edward Memorial has now been subscribed. Part of the sum is to be devoted to the erection of a home for the Queen's Nurses.

Bristol.—The new Royal Infirmary will contain 180 beds and three operating theatres; Mr. Percy Adams, F.R.I.B.A., is architect.

Burnley.—Twenty-three thousand pounds is the sum aimed at for carrying into effect proposals for the extension of Victoria Hospital, Burnley. The scheme has the threefold object of commemorating the Coronation and the jubilee of the municipality and marking the town's respect for the late King.

Coventry.—The new wing at the Coventry and Warwickshire Hospital will be started at once. Of the £25,000 required, £7,396 has now been subscribed.

Darlington.—Extensions to the Darlington Fever Hospital, costing about £850, are contemplated.

Darnley.—A new smallpox hospital is proposed within the grounds of the Darnley Fever Hospital. It is to be of the Spiers Pavilion type, and to cost about £2,000.

Ipswich.—Plans for the King Edward Memorial Sanatorium have been prepared by Mr. H. Munro Cautley and tenders will presently be invited.

### COMING EVENTS OF THE WEEK.

[Communications for this column must be sent to 29 Southampton Street, Strand, before noon on Wednesday.]

Tuesday, April 4.—Tuberculosis Exhibition: Opening ceremony, Campden Technical Institute, Lancaster Road, W., at 12.

Princess Christian presides at the annual meeting of the Ladies' Association of the Hospital for Women, Soho Square, at 31 Great Cumberland Place, at 12.

Nursing Exhibition, Royal Horticultural Hall (four days).

South Wales Nursing Association: Annual meeting at Neath.

Wednesday, April 5.—The Queen visits the National Industrial Home for Crippled Boys, Woolsthorpe House, Wright's Lane.

Tuberculosis Exhibition opens free to the public, Campden Technical Institute, Lancaster Road, W., at 12.

Women's Local Government Society: Performance of Swinburne's *Atalanta in Calydon*, in aid of the funds, Lyceum Theatre, at 3.

Thursday, April 6.—Under the patronage of her Majesty the Queen, her Majesty Queen Alexandra, etc., the annual Sale of Work from institutions connected with the Society for Promoting Female Welfare will be opened by H.H. the Princess Marie Louise of Schleswig-Holstein at the Royal Albert Hall, at 12.30 (two days).

### THE WEEK'S NOVELTIES.

The "JOHN BULL" brands of MILK AND MALT FOODS are well worth a little of the study that has been expended upon them. No. 1, for infants, is a milk-and-malt food, free, as the analysis which accompanies each tin reminds us, of starch or added sugar, and containing carbohydrates, proteins, fat, and mineral matter combined in the form most easily assimilated. No. 2, malted cereal food, is designed for infants over five months old and for invalids generally, the constituents this time including starch, being prepared for a less delicate stomach. The firm, whose address is St. Neots, Huntingdonshire, have also prepared a concentrated food known as "Maltcon," which, of a meaty character, is a cereal diet and claims a higher nutritive value than that of any meat extract.

### APPOINTMENTS VACANT.

Full particulars of these vacancies can be obtained on reference to our advertisement columns:

Birmingham and Midland Homœopathic Hospital and Dispensary.—House Surgeon.

Coventry and Warwickshire Hospital.—Junior House Surgeon.

Manchester Royal Infirmary.—Director of Cancer Research Laboratory.

Seamen's Hospital Society.—Two House Physicians, Two House Surgeons.

South Africa.—Assistant Resident House Surgeon.

West Bromwich District Hospital.—Senior House Surgeon.

Western General Dispensary.—Honorary Surgeon.

West Ham and Eastern General Hospital.—House Surgeon, House Physician.

Messrs. Eyre and Spottiswoode, King's Printers, 33 Paternoster Row, have arranged with Messrs. Novello and Co. for the exclusive right to reproduce Sir Frederick Bridge's Coronation Anthem in their souvenir editions of the Book of Common Prayer with Hymns Ancient and Modern. The books will also contain the Authorised Coronation Service to be used in Westminster Abbey on June 22, together with portraits of their Majesties King George V. and Queen Mary. The volumes will be issued at prices ranging from 1s. to 7s.

